# Detection of melon necrotic spot virus by one-step reverse transcription loop-mediated isothermal amplification assay

**DOI:** 10.1371/journal.pone.0230023

**Published:** 2020-03-05

**Authors:** Ning Qiao, Huijie Dai, Jie Liu, Xiaoping Zhu, Jintang Li, Dezhen Zhang, Yongguang Liu

**Affiliations:** 1 College of Plant Protection, Shandong Agricultural University, Taian, Shandong, China; 2 Facility Horticulture Laboratory of Universities in Shandong, Weifang University of Science and Technology, Shouguang, Shandong, China; University of Georgia, UNITED STATES

## Abstract

Melon necrotic spot virus (MNSV) can cause significant economic losses due to decreased quality in cucurbit crops. The current study is the first to use reverse transcription loop-mediated isothermal amplification (RT-LAMP) for detection of MNSV. A set of four LAMP primers was designed based on the coat protein gene sequence of MNSV, and a RT-LAMP reaction was successfully performed for 1 h at 62°C. The results of RT-LAMP showed high specificity for MNSV and no cross-reaction with other viruses. Compared to traditional reverse transcription-PCR (RT-PCR), the RT-LAMP assay was 10^3^-fold more sensitive in detecting MNSV. Due to its sensitivity, speed and visual assessment, RT-LAMP is appropriate for detecting MNSV in the laboratory.

## Introduction

The quality of melons can be affected by the harmful pathogen melon necrotic spot virus (MNSV), which results in significant losses to the economy of producer countries [1]. Research into the disease caused by MNSV has been reported extensively [2–7] and the first case of the disease in China was reported in 2008 [8].

MNSV belongs to the genus *Carmovirus* of the family Tombusviridae [9]. Host selection is relatively specific, with species representing only part of the Cucurbitaceae family acting as hosts [10]. The virus can cause necrosis of roots, leaves and fruits, from which the virus is transmitted mainly by the chytrid fungus *Olpidiumbornovanus* in nature [11–14]. In addition, MNSV can also be spread through mechanical inoculation [7] and propagation *via* infected seeds [10, 15].

The MNSV genome containing at least five open reading frames (ORFs) such as p29, p89, p7a, p7b, and p42 consists of a 4.3-kb positive single-stranded RNA chain [16, 17].

Conventional detection of MNSV is performed by enzyme-linked immunosorbent assay (ELISA) or reverse transcription–polymerase chain reaction (RT-PCR) [18], but these methods have the disadvantages of taking a long time to complete, and of requiring the availability of specialized equipment and expensive consumables. Recent research has used the reverse transcription-loop-mediated isothermal amplification (RT-LAMP) to detect plant [19, 20], animal [21, 22] and human viruses [23, 24]. This method has been shown to exhibit high sensitivity, reliability and rapid completion time; furthermore, it requires cheaper consumables and simpler equipment than the conventional RT-PCR detection method. This paper focuses mainly on the practical value of rapid detection of MNSV by RT-LAMP.

## Materials and methods

### Source of materials

Scientific researchers from Shandong Agricultural University and Weifang University of Science and Technology were allowed to collect and test samples during September 2018 to May 2019 in the company (Shouguang Wanglin Agricultural Development Co., Ltd., China)’s vegetable planting bases such as Shouguang and Changle districts of Shandong Province, China.

We collected symptomatic virus-infected melon and cucumber leaves from Shouguang and Changle, respectively, in Shandong Province, China. Viruses present were identified by RT-PCR and sequencing as MNSV and cucurbit chlorotic yellows virus (CCYV) (from melon), cucumber mosaic virus (CMV) and cucumber green mottle mosaic virus (CGMMV) (from cucumber), and healthy leaves from melon were stored in the laboratory (S1 Table).

### Design of RT-LAMP primers

The inner (MNSV-FIP and MNSV-BIP) and the outer primers (MNSV-F3 and MNSV-B3) constituted a set of RT-LAMP primers, which were designed based on the conserved sequences of MNSV coat protein gene (GenBank accession number EU016217,DQ922807,GU480022,KT923150,KY124137,AB044292,AB106106,AF488692,MK604924,KR094068) and using Primer Explorer V5.0 (http://primerexplorer.jp/lampv5e/index.html) (S1 Fig). For conventional RT-PCR amplification, specific primers MNSV-F and MNSV-R were designed (Table 1 and S1 Fig). All primers were synthesized by Invitrogen (Shanghai, China).

**Table 1:** Primers designed for RT-LAMP and PT-PCR of MNSV.

Primer names	Length (bp)	Position	Sequence(5’-3’)	Application
MNSV-F3	18	397–414	TCGGCTCCGTTTCACCTA	RT-LAMP
MNSV-B3	20	601–620	ACTTCCGGTCGACAGCATTA	
MNSV-FIP	42	470–491,425–444	TGGGGAGGGGGTCTTGTGAATC-ACCGGATCTACTTCCACTGG	
MNSV-BIP	42	517–538,567–586	TGCTCATTACGCTGACTCAGCG-CCTCCACGTATTGTCACACG	
MNSV-F	18	38–55	GCTATAGATGTGGTTCCT	RT-PCR
MNSV-R	20	581–600	GTATCATTCATGTACCTCCA	

### Extraction of total RNA

Total RNA was extracted from MNSV-infected naturally melon leaves and healthy melon leaves using the MiniBEST Universal RNA Extraction Kit (TaKaRa). The concentration of total RNA was determined using a micro-volume UV spectrophotometer (Nanodrop 2000; Thermo Fisher Scientific) and the RNA samples were then stored at -80°C prior to carrying out RT-LAMP and RT-PCR assays.

### Initial establishment of RT-LAMP

The RT-LAMP reaction was performed in a 25 μL reaction mixture containing 1.6 μM of each of the MNSV-FIP and MNSV-BIP primers, 0.2 μM of the MNSV-F3 and MNSV-B3 primers, 12.5 μL of 2 x RT-LAMP reaction buffer [40 mM Tris-HCl (pH 8.8), 20 mM KCl, 20 mM (NH_4_)_2_SO_4_,16 mM MgSO_4_, 0.2% Triton X-100, 2.4 mM dNTP, 1.6 M betaine] (Sigma–Aldrich), 1 μL of *Bst* DNA polymerase (8 U/μL, New England Biolabs), 1 μL of M-MLV reverse transcriptase (200 U/μL, Promega), 1 μL of RNasin^®^ribonuclease inhibitor (40 U/μL, Promega), 2 μL of RNA template (replaced by RNA of healthy melon leaves for the negative control and ddH_2_O for the blank control) and RNase/DNase-free ddH_2_O.

The RT-LAMP reaction mixture was heated for 1 h at 61°C and the reaction was terminated by heating for 10 min at 80°C. Aliquots (5 μL) of the products of RT-LAMP amplification were verified using 1.5% agarose gel electrophoresis (DL 2000 DNA marker (100–2000 bp); TaKaRa). Positive results could be translated into a fluorescent color change which could be observed under UV light at 365 nm by adding 0.2 μL SYBR Green I (10×, Invitrogen) to the post-reaction solution, or into a visual color change by adding 2 μL of 60 μM hydroxynaphthol blue (HNB; Sigma–Aldrich) to the pre-reaction solution, the latter change in color (from violet to sky blue) being clearly observable with the naked eye.

In order to optimize the reaction conditions, different reaction temperatures of 60°C, 61°C, 62°C or 63°C were carried out for 1 h, and different reaction times of 30, 45, 60 or 70 min at 62°C were tested.

### Specificity analysis of RT-LAMP assay

The total RNA from leaf tissues infected naturally by CGMMV, CMV or CCYV was extracted by the same method. Total RNA from plants infected with these viruses and MNSV was used as templates in the RT-LAMP assay under the optimized conditions determined in the current study.

### Sensitivity analysis of RT-LAMP and RT-PCR assays

The relative sensitivities of RT-LAMP and RT-PCR were assessed using 10-fold serial dilutions of the MNSV total RNA as templates. Using the PrimeScript II 1st Strand cDNA Synthesis Kit (TaKaRa) with the reverse primer (MNSV-R), reverse transcription was carried out according to the manufacturer’s instructions. A PCR reaction mixture of 25 μL contained 2.5 μL of cDNA, 0.2 μM each of MNSV-F and MNSV-R primers and 2x *Taq* PCR MasterMix (Tiangen), with thermocycling conditions of 94°C for 5 min, 30 cycles of 94°C for 30 s, 55°C for 30 s, and 72°C for 30 s, with a final extension of 72°C for 10 min.

### Applicability of RT-LAMP assay

A total of 24 samples of melon leaves suspected of being naturally infected with MNSV (on the basis of characteristic symptoms) were collected from various commercial melon greenhouses in the ShandongProvince area and RNA was extracted from each sample. RT-PCR and the optimized RT-LAMP methods were used for sample detection, with the amplification products being observed by electrophoresis and color/fluorescence indicator dyes.

## Results

### Establishment of optimal reaction conditions for RT-LAMP

**Fig 1:**
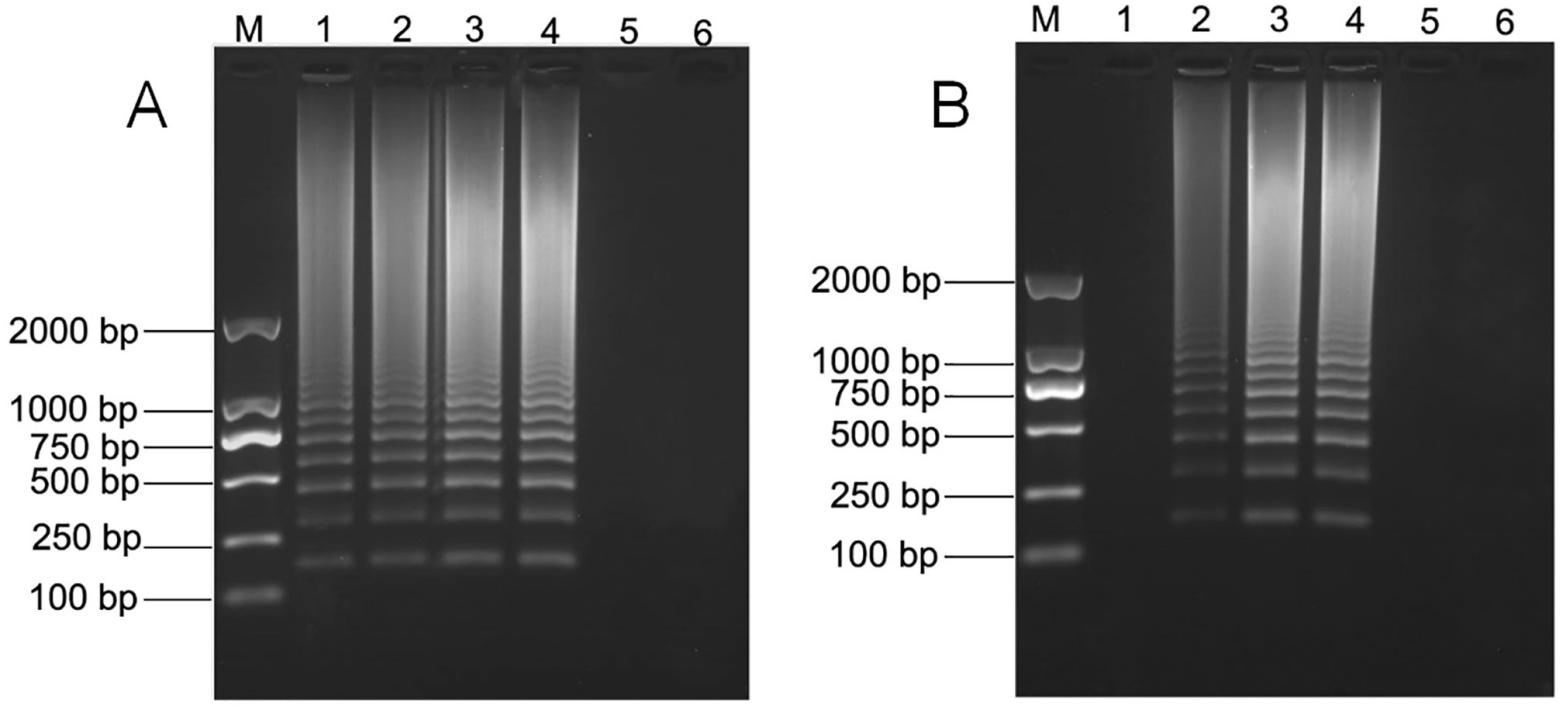
Optimization of RT-LAMP reaction conditions for MNSV. (A) Effects of reaction temperature on the RT-LAMP reaction: lanes 1, 2, 3, and 4 corresponded to amplification temperatures at 60, 61, 62, and 63°C, respectively. (B) Effect of reaction time on the RT-LAMP reaction: lanes 1, 2, 3, and 4 corresponded to 30, 45, 60, and 70 min, respectively. Lane M corresponded to DL 2000 DNA marker (100–2000 bp), lanes 5, and 6 corresponded to negative control and blank control.

Different temperatures in the range 60–63°C were tested to optimize the reaction temperature for RT-LAMP, under which the ladder of bands of different sizes could clearly be distinguished by agarose gel electrophoresis (Fig 1A). With regard to the intensity of the bands obtained, 62°C was selected as the optimal reaction temperature, on the basis that band intensity was the strongest at that temperature. When the reaction time reached 60 min or more (up to 70 min), the intensity of the product of RT-LAMP was the clearest and tended to be stable. The optimal time at a reaction temperature of 62°C was determined to be 60 min (Fig 1B).

### Specificity of RT-LAMP assay for MNSV

**Fig 2:**
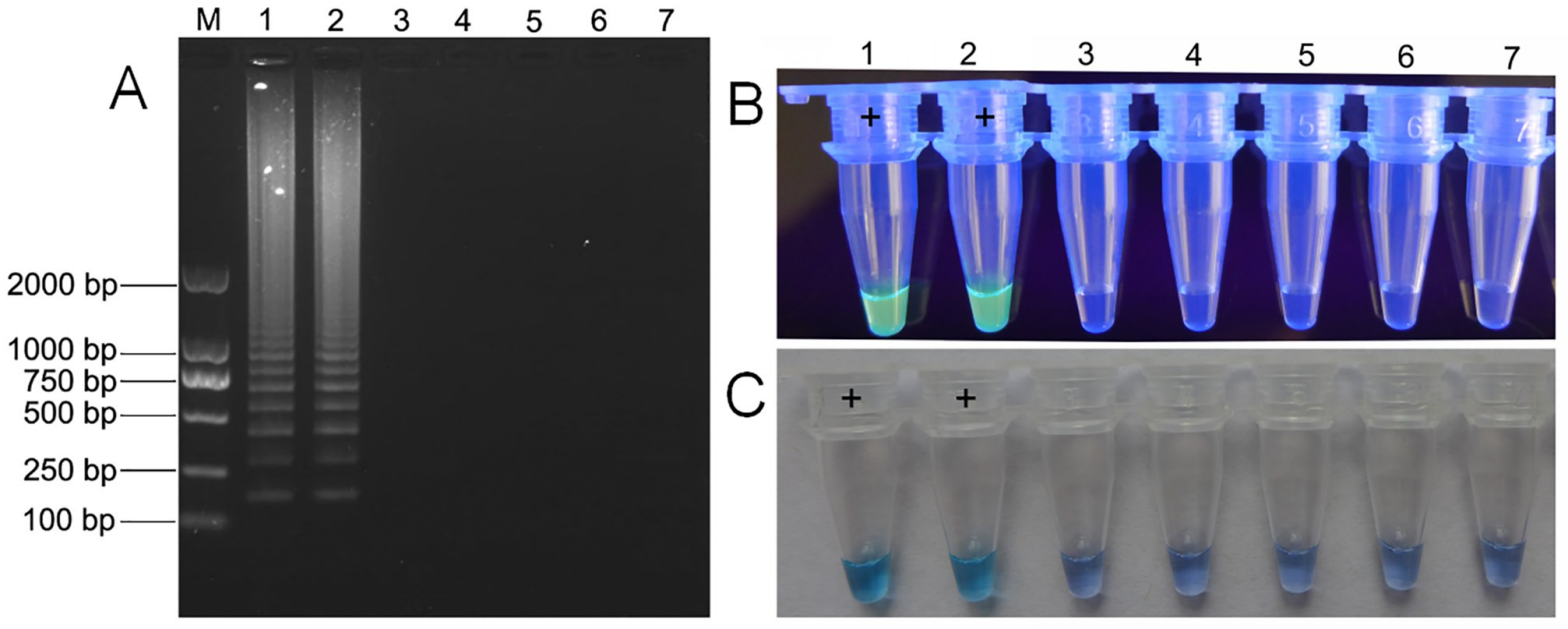
Specificity of RT-LAMP reaction conditions for MNSV. (A) Specificity analysis of RT-LAMP by 1.5% agarose gel electrophoresis. Lane M, DL 2000 DNA marker; lane 1, MNSV-infected melon leaf from Shouguang, China; lane 2, MNSV-infected melon leaf from Changle, China; lane 3, CGMMV-infected cucumber leaf; lane 4, CMV-infected cucumber leaf; lane 5, CCYV-infected melon leaf; and lanes 6–7, negative control and blank control. (B) Visualization of the RT-LAMP following staining with SYBR Green I under UV light. (C) Visualization of the RT-LAMP by the naked eye with the color change of hydroxynaphthol blue from violet (negative) to sky blue (positive).

RNA templates extracted from cucumber and melon leaves infected by other viruses were used to test the specificity of RT-LAMP for MNSV. Under the optimized reaction conditions, electrophoresis analysis showed that only the ladder of bands was observed in samples infected with MNSV, whereas the other samples gave negative results (Fig 2A). Meanwhile, when SYBR Green I (post-reaction) or HNB (pre-reaction) were added into individual samples, the fluorescence and sky blue, respectively, appeared only in samples infected by MNSV (Fig 2B and 2C).

### Sensitivity comparison of RT-LAMP with RT-PCR

**Fig 3:**
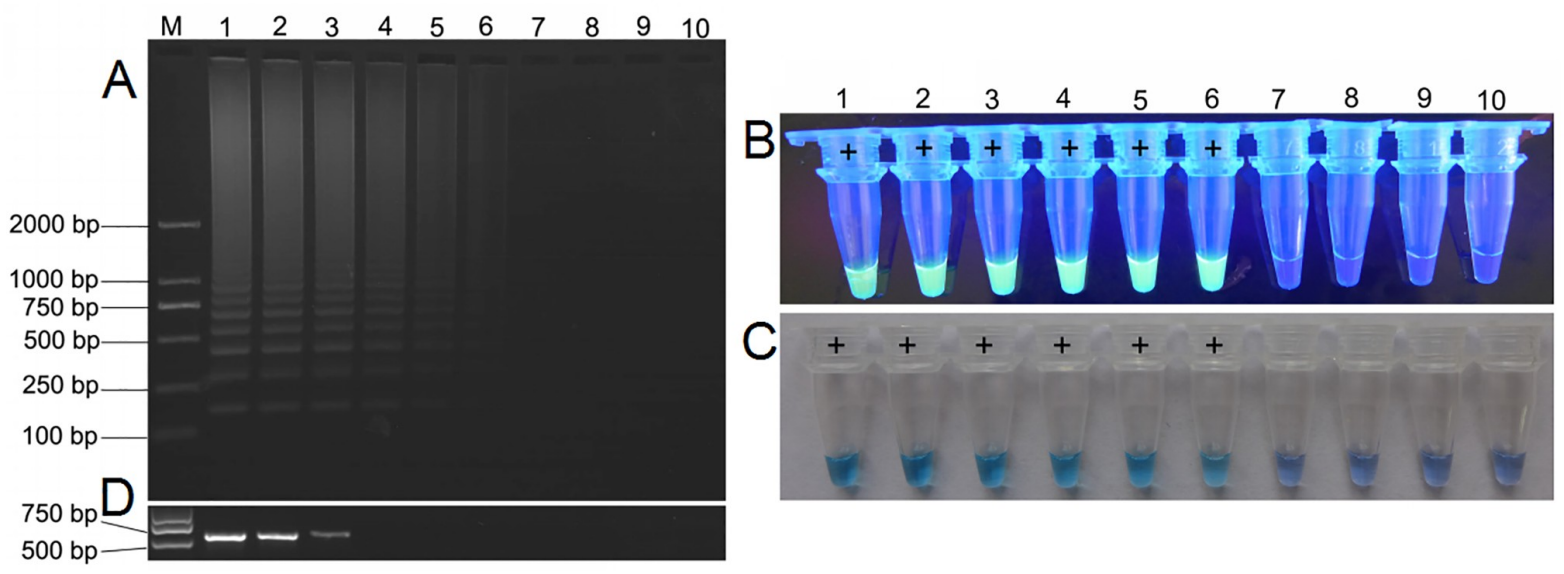
Comparison of the sensitivity of RT-LAMP and RT-PCR assays for MNSV. (A) Sensitivity analysis of the RT-LAMP by agarose gel electrophoresis using a serial dilution of total RNA. Lane M, DL 2000 DNA marker (100–2000 bp); lanes 1–8, from 7.5×10^−1^ to 7.5×10^−8^ μg/μL of total RNA extracted from MNSV-infected melon leaves per assay; and lanes 9–10, negative control and blank control. Visualization of the RT-LAMP assay according to (A) with addition of SYBR Green I under UV light (B) or the color change of hydroxynaphthol blue (C). (D) Agarose gel illustrating the RT-PCR products from the same dilution series of total RNA as in lanes 1–10 in (A).

In a comparison of RT-LAMP with RT-PCR, the detection limits were determined using 10-fold serial dilutions of total RNA (7.5×10^−1^ to 7.5×10^−8^μg/μL) from MNSV-infected melon leaves. At a dilution of 7.5×10^−6^μg/μL, a positive reaction was observed from RT-LAMP amplification (Fig 3A, 3B and 3C), whereas the lowest dilution was 7.5×10^−3^μg/μL at which the 563bp target fragment of RT-PCR (Fig 3D) was amplified. The sensitivity of RT-LAMP for MNSV was 10^3^-fold greater than that for RT-PCR, which also took about 2 h to amplify and detect, compared with 1 h for RT-LAMP.

### Detection of MNSV from diseased samples

**Fig 4:**
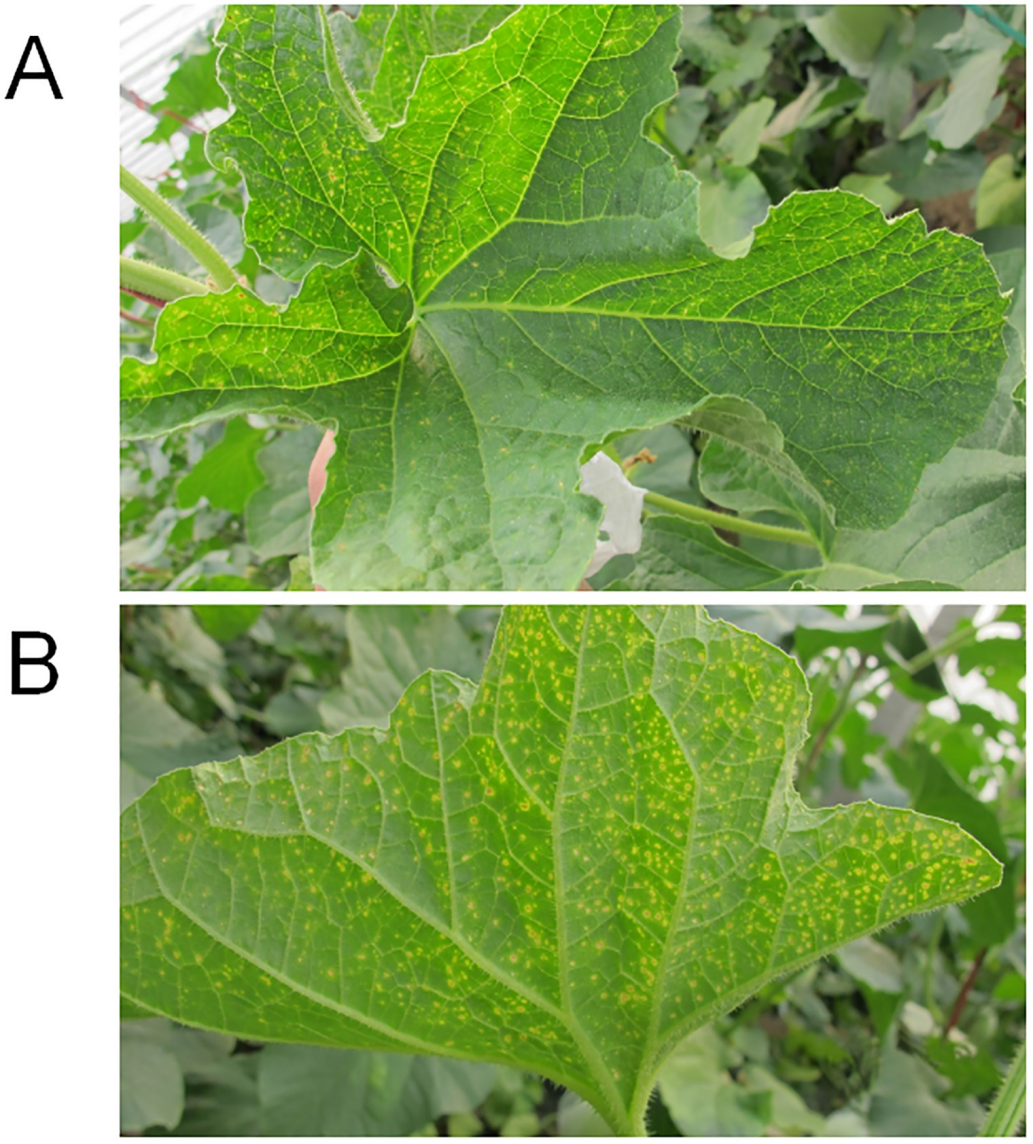
Melon leaf symptoms of MNSV infection. (A) and (B)Symptoms of front and back MNSV-infected naturally melon leaves.

**Fig 5:**
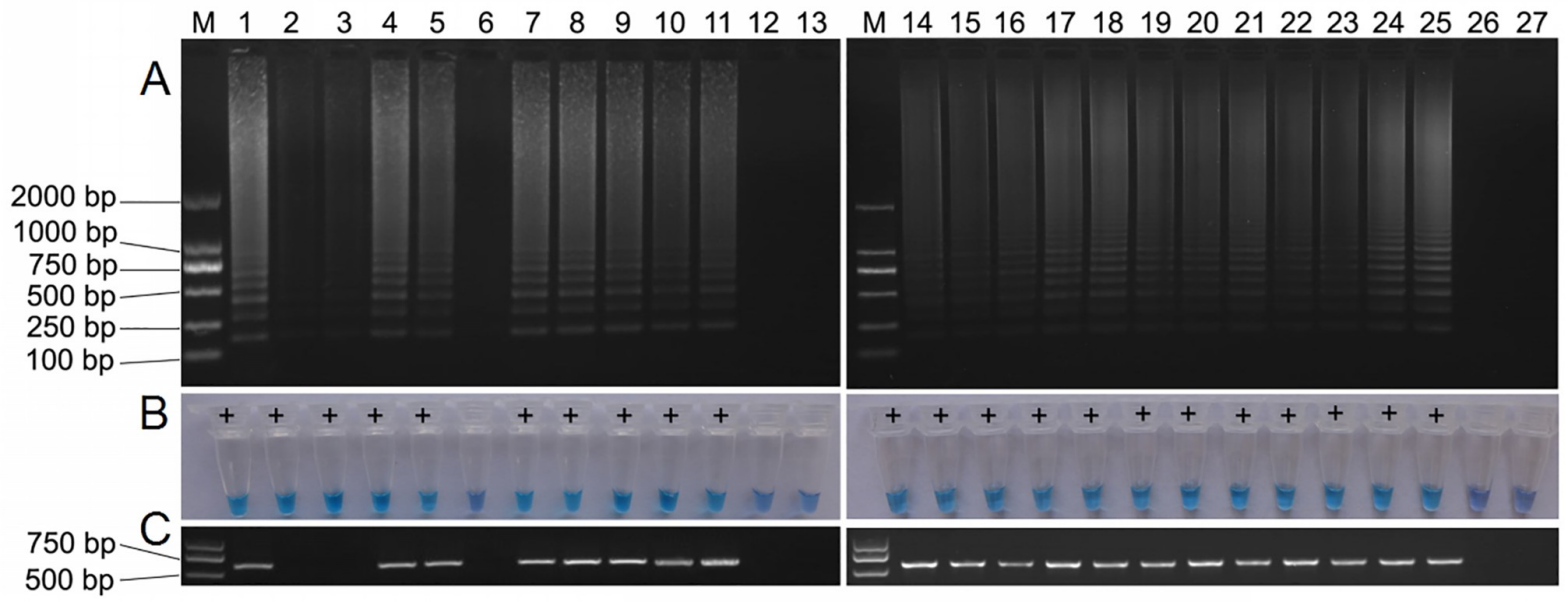
Use of RT-LAMP assay to detect MNSV. (A) Agarose gel illustrating the RT-LAMP products from 24 melon leaf samples in lanes 1–24; lane 25, positive control; and lanes 26–27, negative control and blank control. (B) Visualization of the RT-LAMP assay according to 24 samples with the color change of adding hydroxynaphthol blue. (C) Agarose gel illustrating the RT-PCR products from 24 melon leaf samples in lanes 1–24; lane 25, positive control (MNSV samples that had been detected above); and lanes 26–27, negative control and blank control.

Twenty-four melon leaves with symptoms typical of MNSV infection (Fig 4) were collected and assessed for the presence of MNSV using RT-LAMP and RT-PCR. The results showed that 21 of the 24 samples tested positive using the RT-LAMP assay, representing good feasibility and applicability of the samples detection by agarose gel electrophoresis and visualization of RT-LAMP (Fig 5A and 5B). Positive results of 19 samples by conventional RT-PCR were due to the fact that two samples were not detected compared to RT-LAMP (Fig 5C). In addition, three of these samples could not be detected by either method, which may confirm that these samples were not infected by MNSV.

## Discussion

Related research has shown that MNSV can infect many cucurbitaceous plants in a number of countries [18], making it one of the major risk factors for fruit and vegetable production in this plant family. Developing an efficient and convenient method to rapidly detect MNSV is necessary and has stimulated research in this area. In a comparison of real-time PCR, multiplex PCR and RT-PCR [25–27], the products of positive reactions of these assays were achieved through a series of temperature changes using specific instruments. But the emergence of RT-LAMP and Recombinase Polymerase Amplification (RPA) [28, 29], based on isothermal amplification technology, makes sample detection faster, more economical and more operator friendly, as a consequence of the color-based visual results, based on color which provide a qualitative reaction that can be detected by the naked eye. Unfortunately, RPA technology uses a very complex enzyme reaction system resulting in higher detection costs [30] than with RT-LAMP.

The one-step RT-LAMP method described in this study was designed and established to detect MNSV in melon efficiently and effectively. Using either a heated block or a water bath, RT-LAMP for MNSV was performed successfully with the optimal conditions of 62°C and a 60 min reaction time. After adding HNB or SYBR Green I, the DNA band-based positive response could be translated into a color change that could be observed clearly. The RT-LAMP detection was highly specific for MNSV, with none of the other viruses from cucurbitaceous hosts exhibiting any amplification. The current evaluation study proves that the RT-LAMP assay is a reliable method with which to detect MNSV.

Numerous studies have confirmed that the RT-LAMP method has greater sensitivity than either standard RT-PCR or nested RT-PCR [20, 22, 31]. The visual assessment of the RT-LAMP assay can be independent of expensive equipment such as a PCR thermocycler or a metal thermostat-controlled heater. The presence of white turbidity in the RT-LAMP positive reaction is due to the production of a large amount of magnesium pyrophosphate by-product, which can be observed with the naked eye [32, 33]. However, other factors, such as the intensity of light, transparency of the reaction container material, the concentration of the product or a visual error by the observer, can make false positives (based on the accumulation of pyrophosphate) possible [34]. The use of a turbidimeter (e.g. the Loopamp Realtime Turbidimeter) can avoid this problem [35, 36], but requires the purchase of additional, albeit relatively inexpensive, equipment.

The addition of a fluorescent dye after the reaction, such as SYBR Green I, causes bright green fluorescence of the positive product (Figs 2B and 3B), but opening of the tubes to carry out this addition may cause aerosol pollution [34]. By adding hydroxynaphthol blue (HNB) or calcein to the pre-reaction solution, rather than adding SYBR Green I to the post-reaction solution, brings about color changes visible to the naked eye associated with positive reactions to highlight the positive/negative distinction without the need for expensive instrumentation; it would also reduce the risk of contamination by aerosol pollution [34, 37]. However, the color change caused by calcein is from the orange color of the negative reaction to the yellow of the positive reaction, and subjective judgment based on such a small color difference can increase observer error. Therefore, the present study used HNB, which produced consistent experimental results and a clear distinction between positive (sky blue) and negative results (violet) (Fig 5B).

In order to obtain better RT-LAMP reaction conditions, further optimization would be advantageous. Adjustment of Mg^2+^ concentration in the reaction mixture has been reported to further optimize the reaction conditions in previous studies, and the published results have shown that 8 mM Mg^2+^ was the optimum concentration applicable in most situations [33, 38], consequently, Mg^2+^ optimization steps were omittedfrom the current study [39, 40]. Specific Mg^2+^ concentrations, such as 4 mM and 7 mM [41, 42], depending on the virus type, have been used in published RT-LAMP detection assays. So based on previous research results, 8 mM Mg^2+^ was used in this work with consistent and clear outcomes.

In summary, the one-step RT-LAMP assay developed in this study has excellent practical features, achieving rapid, accurate and visible detection of MNSV in the laboratory.

## Supporting information

S1 Fig Multiple sequence alignment of the partial coat protein gene of 10 isolates of MNSV from different countries. The RT-LAMP and RT-PCR primers used in this work were designed based on conserved genome regions (green box). Primer MNSV-FIP was a combination of primers F1c and F2 (5’-3’), Primer MNSV-BIP was a combination of primers B1c and B2 (5’-3’).
(PDF)

S1 Table Primer sequences for detection of CCYV, CMV, CGMMV. (DOC)

S1 File Raw images. (PDF)
