# Supplementary material for: Detection of melon necrotic spot virus by one-step reverse transcription loop-mediated isothermal amplification assay
Source: PLoS One. 2020 Mar 5;15(3):e0230023. doi: 10.1371/journal.pone.0230023 (PMC7058275; doi:10.1371/journal.pone.0230023)
Supplement: S1 Fig — The RT-LAMP and RT-PCR primers used in this work were designed based on conserved genome regions (green box). Primer MNSV-FIP was a combination of primers F1c and F2 (5’-3’), Primer MNSV-BIP was a combination of primers B1c and B2 (5’-3’). (PDF) [file pone.0230023.s001.pdf]

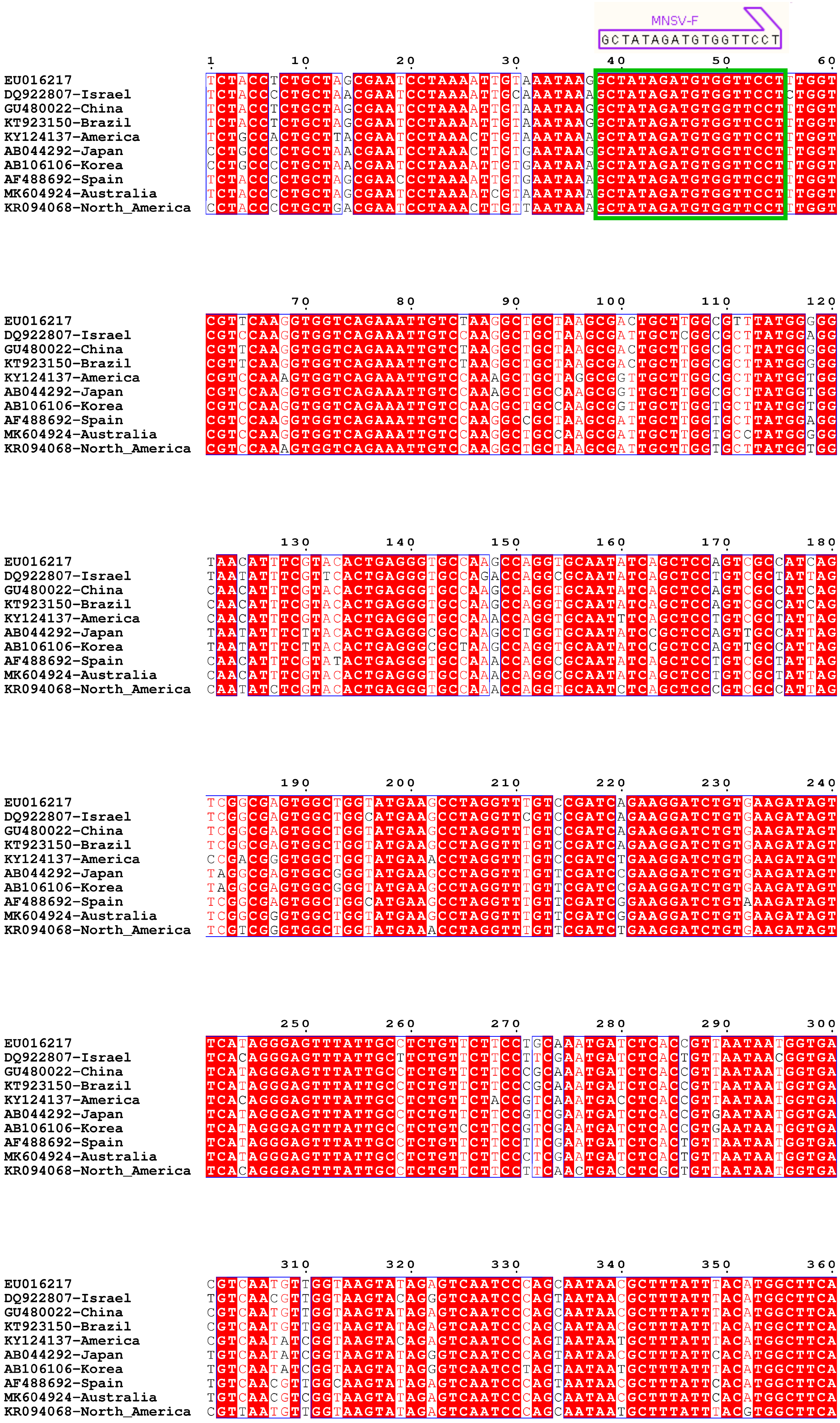
MNSV-F
GCTATAGATGTGGTTCCT
EU016217
DQ922807-Israel
GU480022-China
KT923150-Brazil
KY124137-America
AB044292-Japan
AB106106-Korea
AF488692-Spain
MK604924-Australia
KR094068-North_America

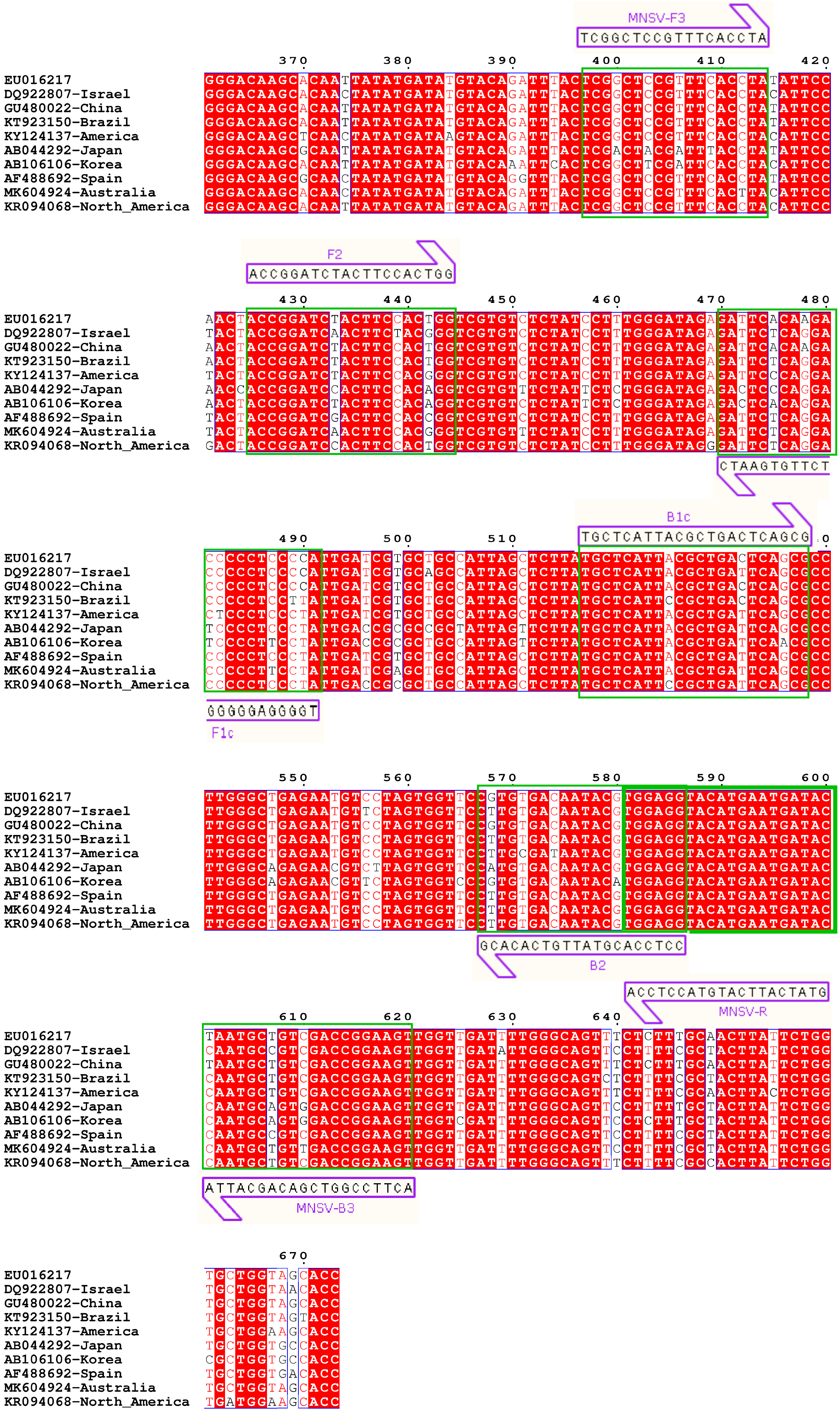

MNSV-F3
TCGGCTCCGTTTCACCTA
F2
ACCGGATCTACTTCCACTGG
CTAAGTGTTCT
B1c
TGCTCATTACGCTGACTCAGCG
GGGGGAGGGGT
F1c
GCACACTGTTATGCACCTCC
B2
ACCTCCATGTACTTACTATG
MNSV-R
ATTACGACAGCTGGCCTTCA
MNSV-B3
EU016217
DQ922807-Israel
GU480022-China
KT923150-Brazil
KY124137-America
AB044292-Japan
AB106106-Korea
AF488692-Spain
MK604924-Australia
KR094068-North_America
