# Supplementary material for: Detection of melon necrotic spot virus by one-step reverse transcription loop-mediated isothermal amplification assay
Source: PLoS One. 2020 Mar 5;15(3):e0230023. doi: 10.1371/journal.pone.0230023 (PMC7058275; doi:10.1371/journal.pone.0230023)
Supplement: S1 Table — (DOC) [file pone.0230023.s002.doc]

**S1 Table Primer sequences for detection of CCYV, CMV, CGMMV.**

| **Primer names** | **Sequence (5’-3’)** | **Length (bp)** | **Tm(**^o^C**)** |
| --- | --- | --- | --- |
| CCYV-F | CGTAAGTGATCGCAATCAAT | 876 | 54 |
| CCYV-R | AGTGATCACTTGACCATCTCC |
| CMV-CPF | ATGGACAAATCTGAATCAACC | 777 | 52 |
| CMV-CPR | TAAGCTGGATGGACAACCCGT |
| CGMMV-F | ATGGCTTACAATCCGATCACACC | 486 | 59 |
| CGMMV-R | CTAAGCTTTCGAGGTGGTAGCCTC |
