# Supplementary figures and images for: Detection of melon necrotic spot virus by one-step reverse transcription loop-mediated isothermal amplification assay
Source: PLoS One. 2020 Mar 5;15(3):e0230023. doi: 10.1371/journal.pone.0230023 (PMC7058275; doi:10.1371/journal.pone.0230023)

Fig1 A

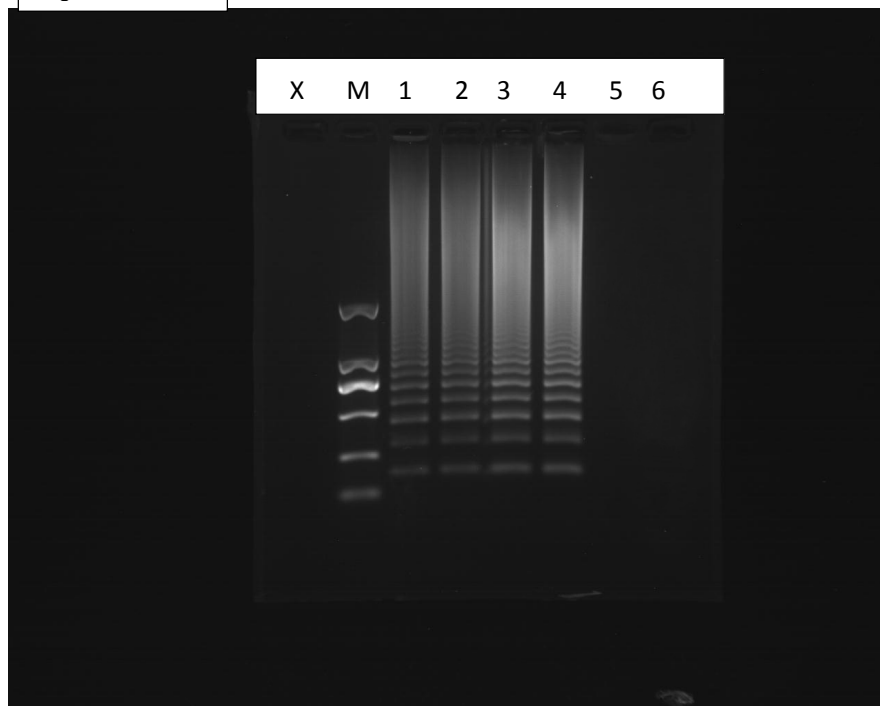

Fig1 B

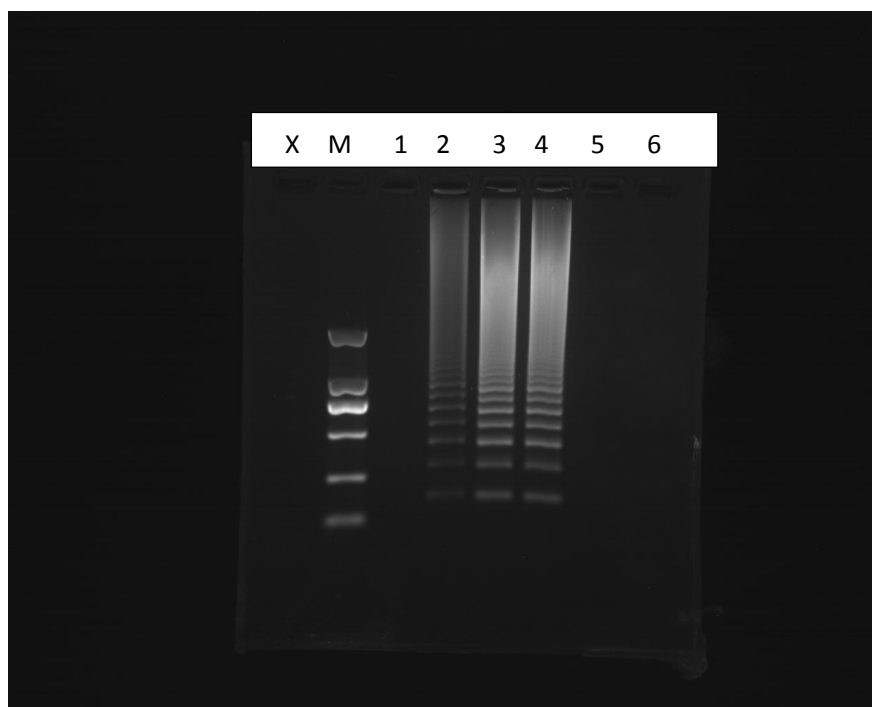

Fig2 A

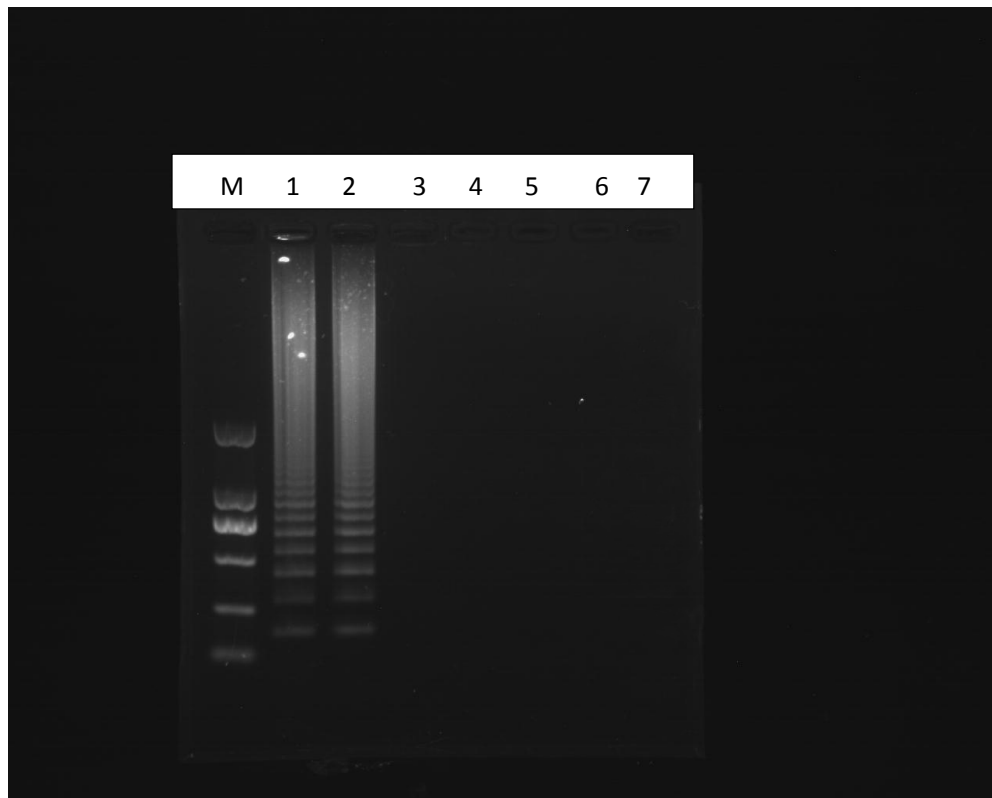

Fig3 A

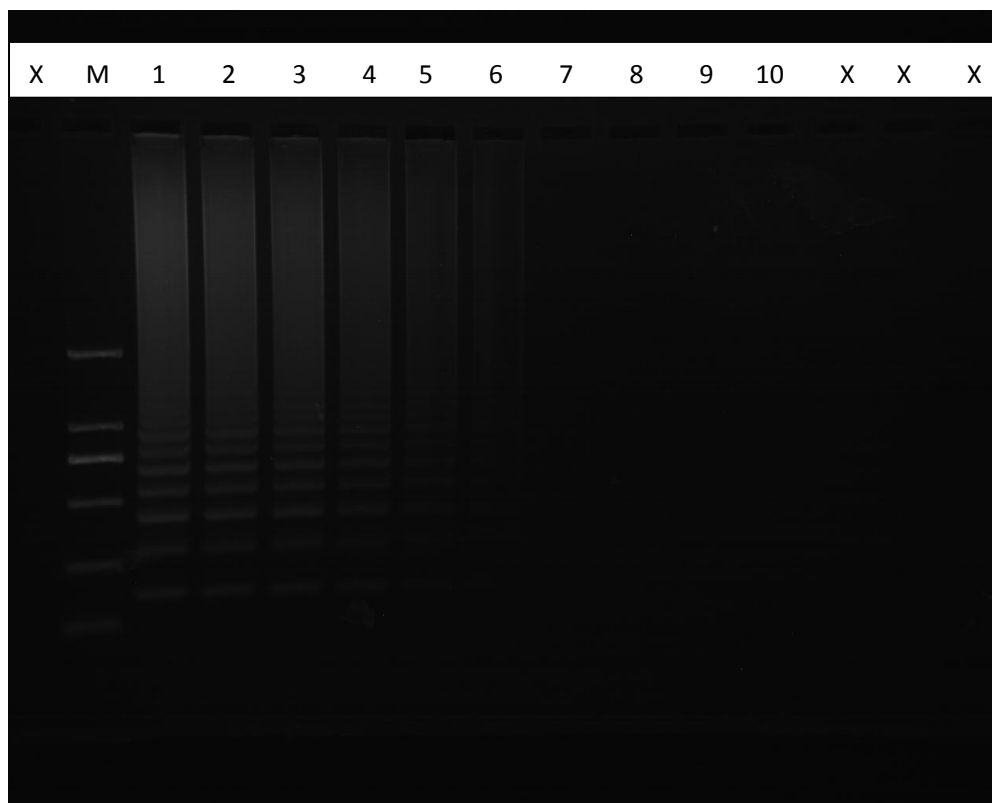

Fig3 D

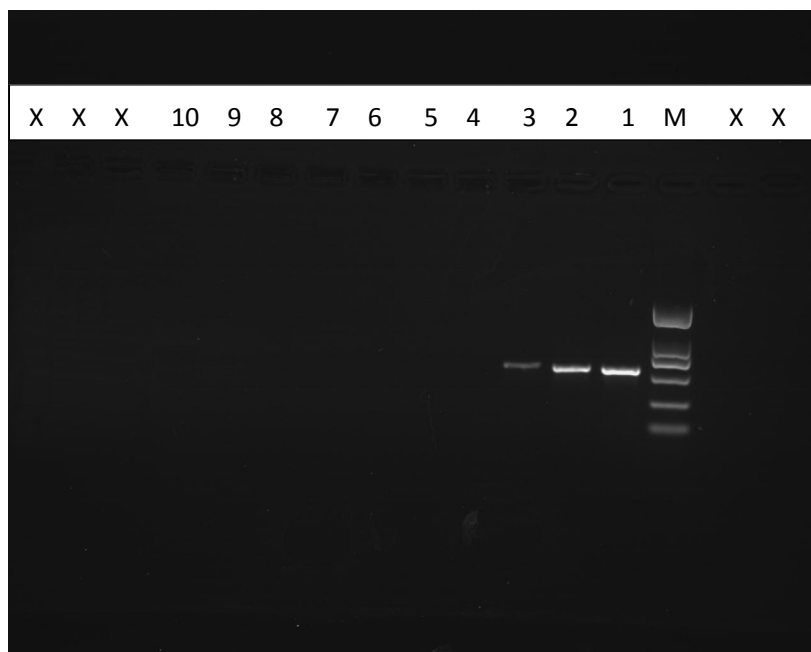

Fig5 A

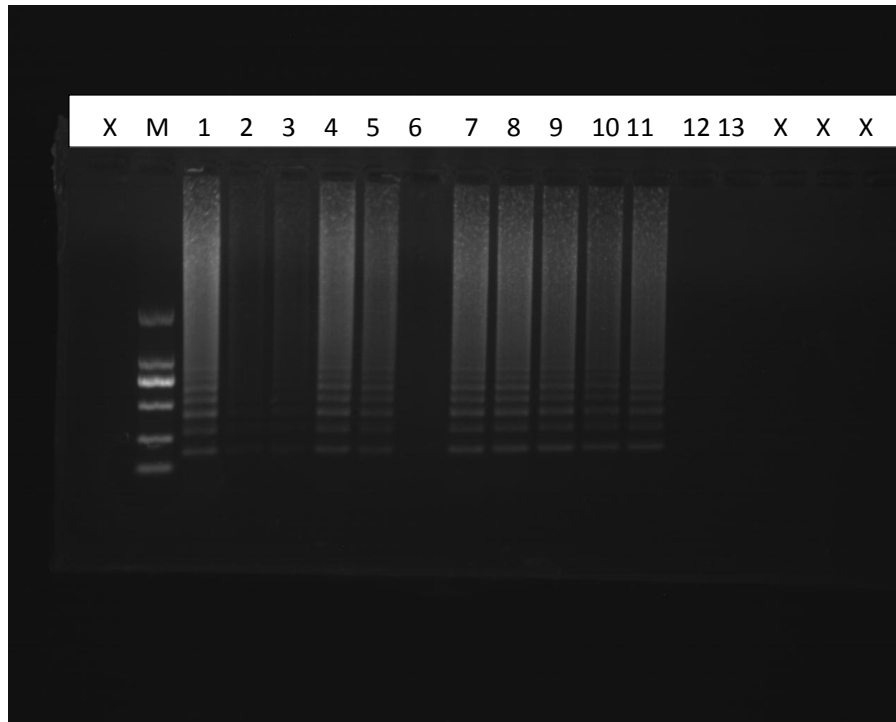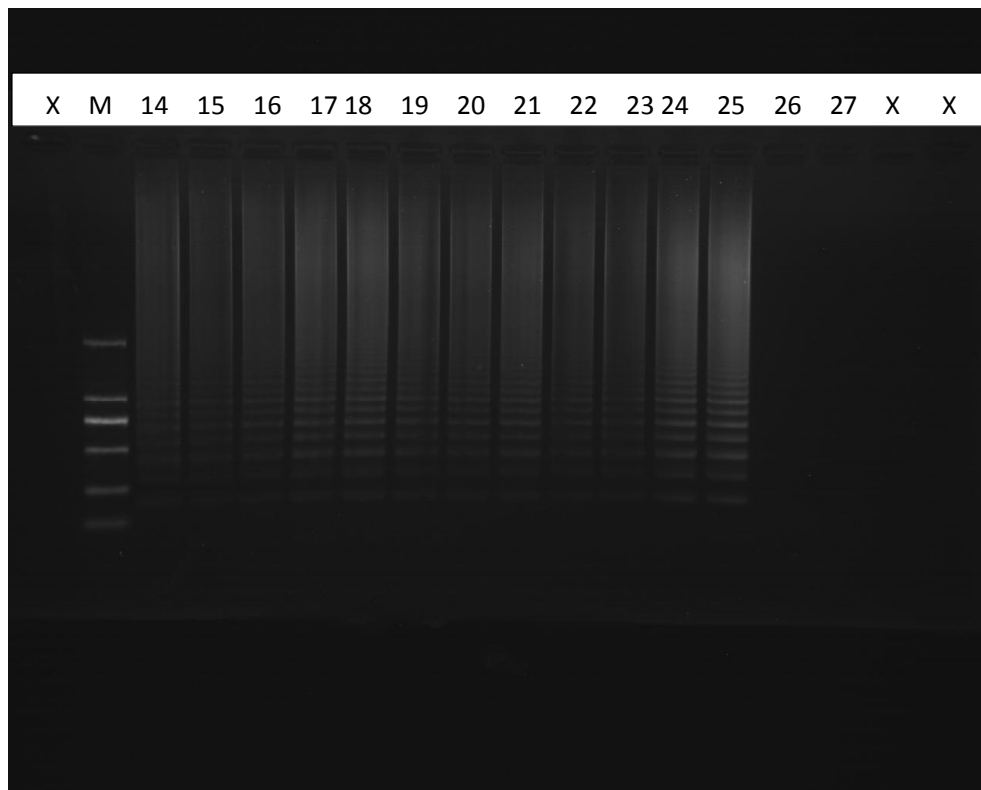

Fig5 C

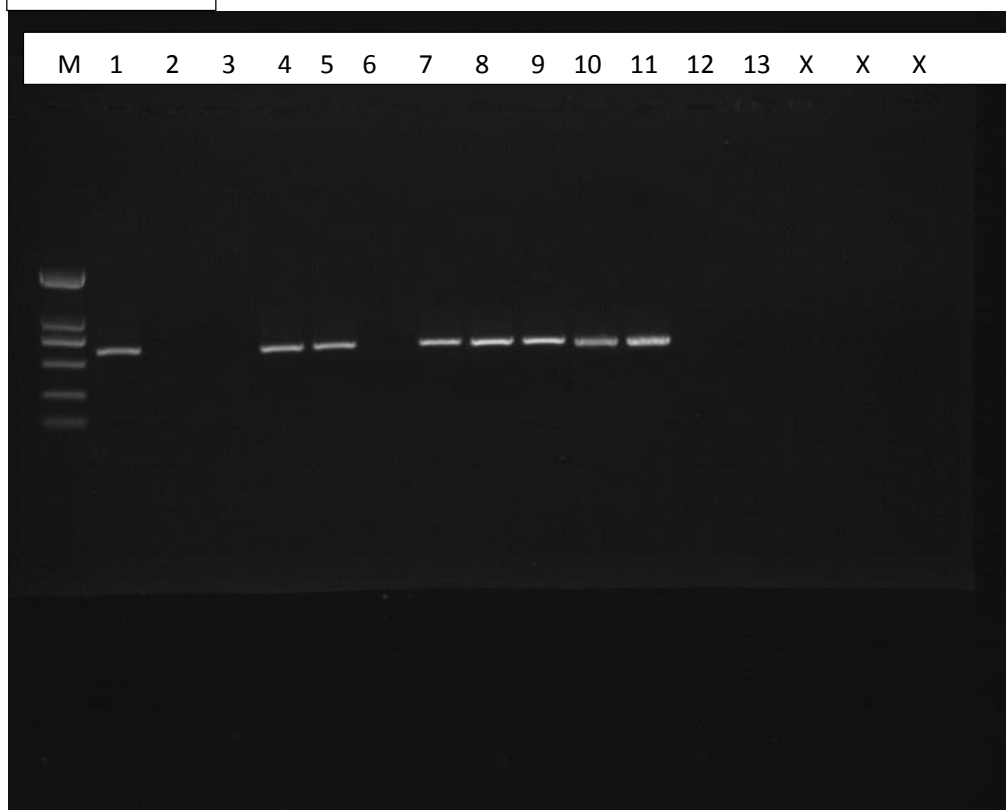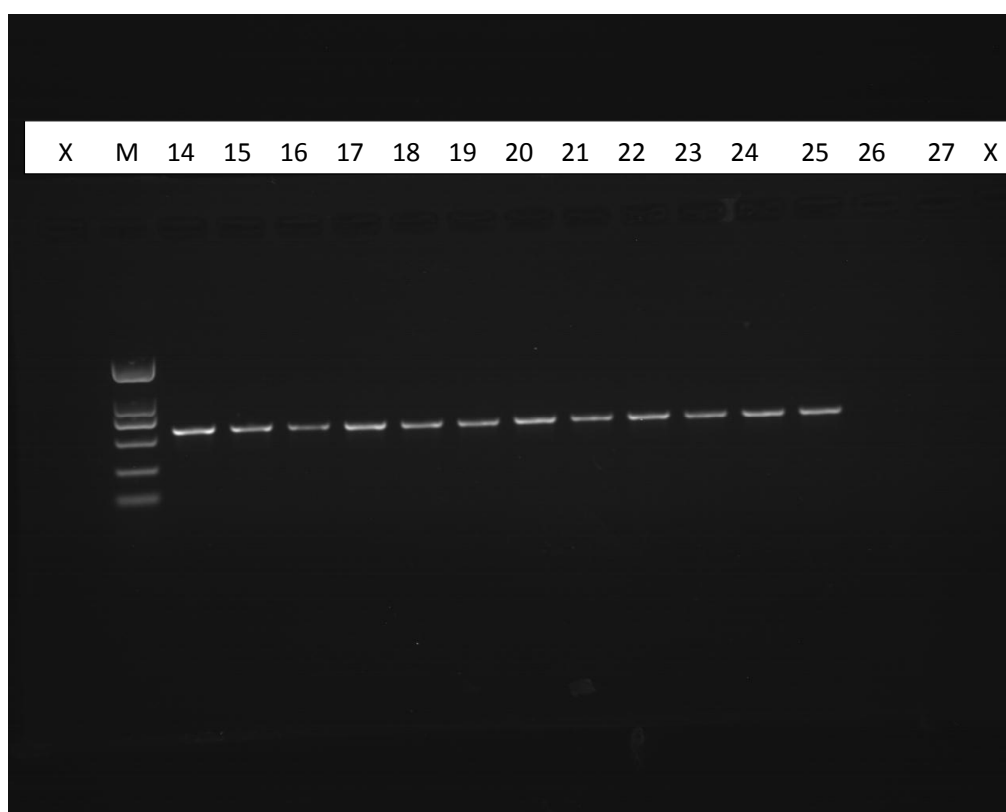

Supplement: S1 File — (PDF) [file pone.0230023.s003.pdf]
